# Successful Treatment of Plasma Cell-Rich Acute Rejection Using Pulse Steroid Therapy Alone: A Case Report

**DOI:** 10.1155/2017/1347052

**Published:** 2017-01-10

**Authors:** Yo Komatsuzaki, Yasuyuki Nakada, Izumi Yamamoto, Mayuko Kawabe, Takafumi Yamakawa, Ai Katsuma, Haruki Katsumata, Aki Mafune, Akimitsu Kobayashi, Yusuke Koike, Hiroki Yamada, Jun Miki, Yudo Tanno, Ichiro Ohkido, Nobuo Tsuboi, Keitaro Yokoyama, Hiroyasu Yamamoto, Takashi Yokoo

**Affiliations:** ^1^Division of Nephrology and Hypertension, Department of Internal Medicine, The Jikei University School of Medicine, Tokyo, Japan; ^2^Division of Urology, The Jikei University School of Medicine, Tokyo, Japan; ^3^Department of Internal Medicine, Atsugi City Hospital, Kanagawa, Japan

## Abstract

Despite the recent development of immunosuppressive agents, plasma cell-rich acute rejection (PCAR) has remained refractory to treatment. Herein, we report an unusual case of PCAR that responded well to pulse steroid therapy alone. A 47-year-old man was admitted for a protocol biopsy three months after kidney transplantation, with a stable serum creatinine level of 1.6 mg/dL. Histological examination showed focal aggressive tubulointerstitial inflammatory cell infiltration of predominantly polyclonal mature plasma cells, leading to our diagnosis of PCAR. Three months following three consecutive days of high-dose methylprednisolone (mPSL) therapy, an allograft biopsy performed for therapy evaluation showed persistent PCAR. We readministered mPSL therapy and successfully resolved the PCAR. Although PCAR generally develops more than six months after transplantation, we diagnosed this case early, at three months after transplantation, with focally infiltrated PCAR. This case demonstrates the importance of early diagnosis and prompt treatment of PCAR to manage the development and severity of allograft rejection.

## 1. Introduction

Plasma cell-rich acute rejection (PCAR) is a distinct morphological form of renal allograft rejection, characterized by the presence of mature plasma cells that comprise more than 10% of the inflammatory cells infiltrating the allograft [1, 2]. While the cause of PCAR remains unknown, the cause is noted in approximately 5–14% of cases of biopsy-proven acute rejection [3]. PCAR is associated with a poor treatment response, resulting in poorer graft survival than seen with other types of acute rejection [1, 2, 4]. Case-control studies have shown that 50% of patients with PCAR lose graft function within six months after transplantation [4]. Although therapies for antibody-mediated rejection (ABMR) and T-cell-mediated rejection (TCMR), as classified at a Banff conference, have been established to an extent, there is no consensus on therapies for PCAR. Here, we report a case of PCAR diagnosed at an early stage (three months after transplantation) that responded well to steroid therapy. This case may provide insight into both the clinicopathological development of PCAR and the characteristics that determine the therapeutic effects and graft loss in PCAR patients.

## 2. Case Report

A 47-year-old Japanese male was admitted to our hospital for a protocol biopsy 3 months after kidney transplantation. He underwent peritoneal dialysis at the age of 41 years due to end-stage kidney disease of unknown origin. At age of 46 years, he received an ABO-compatible living kidney, transplanted from his father (graft weight: 234 g; warm ischemic time: 50 s; cold ischemic time: 1 h 19 min 3 s). Both the recipient and donor were seropositive for cytomegalovirus.

**Figure 1:**
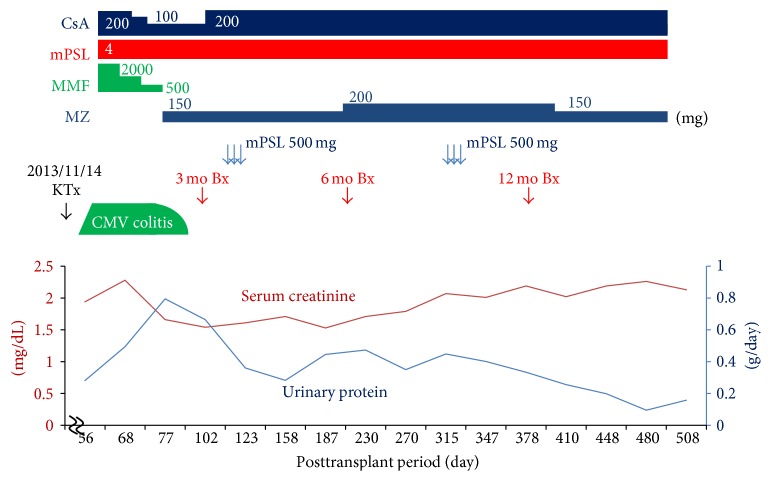
Clinical course. CsA: cyclosporine; MMF: mycophenolate mofetil; mPSL: methylprednisolone; MZ: mizoribine; CMV: cytomegalovirus; KTx: kidney transplantation; Bx: kidney biopsy.

Immunosuppressive therapy consisted of tacrolimus, mycophenolate mofetil (MMF), methylprednisolone (mPSL), and basiliximab. There were no surgical complications. The allograft had slight early dysfunction with serum creatinine level (S-Cr) of 1.94 mg/dL following transplantation, but S-Cr level was kept without a functional exacerbation. The 1 h and 12-day protocol allograft biopsies showed no evidence of rejection. The clinical course of the patient is shown in Figure 1.

One month after transplantation, the patient's ultrasound cardiography examination revealed diffuse hypokinesis (ejection fraction: 35.4%). Because tacrolimus-related hypertrophic myopathy was considered as a possible cause of reduced cardiac function, the calcineurin inhibitor was switched from tacrolimus to cyclosporine A (CsA). Two months after transplantation, the patient was admitted to our hospital with fever, diarrhea, and elevated S-Cr (2.28 mg/dL). Although no pathological findings in the colon mucosa were observed, we diagnosed the patient with cytomegalovirus (CMV) colitis because of a strongly positive CMV pp65 antigenemia (CMV C10/11: 152 and 157 positive cells/slide). We immediately started antiviral treatment with ganciclovir (GCV) and reduced the doses of MMF and CsA. The CMV colitis persisted, however, and the serum CMV antigenemia was not reduced (C10/11: 2356, and 3072 positive cells/slide at peak value). We next administered foscarnet and switched MMF to mizoribine, which has been reported to have antiviral effects in GCV-resistant CMV infections. As a result, the clinical symptoms of CMV colitis immediately disappeared, and the patient's serum CMV antigen was negative six days after foscarnet administration. He was discharged with improved kidney function (S-Cr 1.68 mg/dL) 13 days after administration of foscarnet.

**Figure 2:**
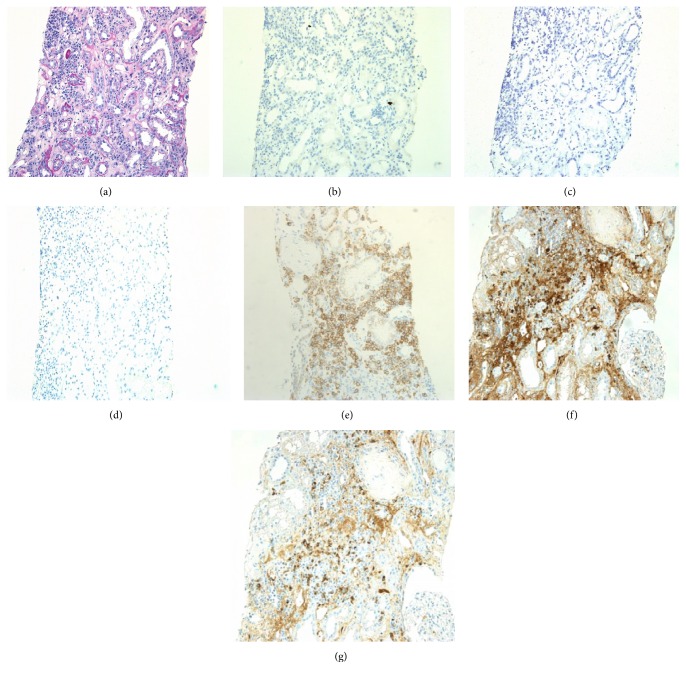
Light microscopic findings for a 3-month protocol biopsy specimen. (a) Mononuclear inflammatory cell infiltration was seen in the interstitial lesions, with plasma cells accounting for approximately 20%. Moderately developed tubulitis was noted in the middle of the field (PAS, ×200). (b–d) Negative staining of SV40 (b), EBER (c), and IgG4 (d) in allograft specimen (×200). (e) CD138-positive interstitial inflammatory cells (×200). (f, g) Infiltrating plasma cells positive for both kappa (f) and lambda (g) light chains, indicating that they are polyclonal (×200).

**Figure 3:**
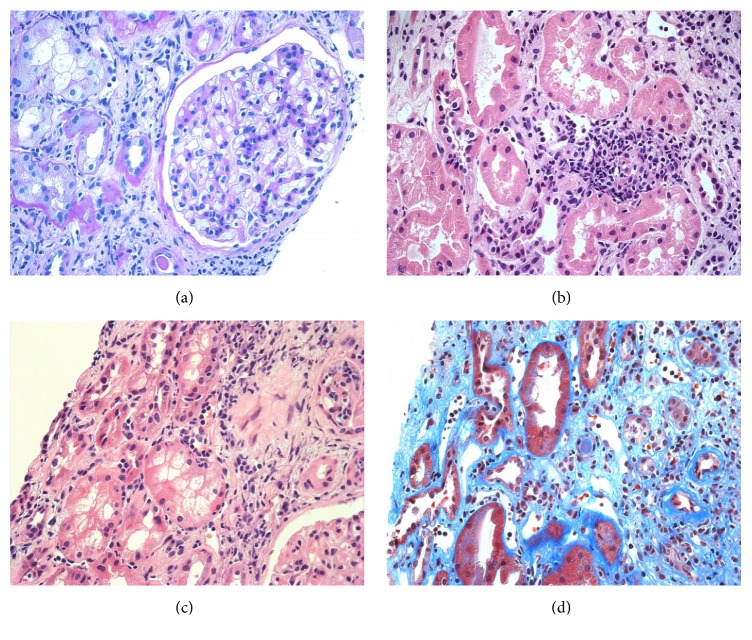
Light microscopic findings in a second biopsy at 3 months after the first antirejection therapy. (a) Transplant glomerulitis, mild (PAS, ×100), (b, c) mild infiltration of inflammatory cells including plasma cells; and mildly developed tubulitis was noted (HE, ×400 (b), ×200 (c)). (d) Peritubular capillaritis was moderately developed (Masson's Trichrome, ×400).

A first protocol allograft biopsy, performed three months after kidney transplantation, incidentally showed focal aggressive interstitial mononuclear cell infiltration, with mild interstitial fibrosis and tubular atrophy and moderate tubulitis (Figure 2(a)). To evaluate these mononuclear cell infiltrates, immunohistochemical analyses of CD138, kappa and lambda light chains, SV40, and IgG4 were performed using monoclonal antibodies. These examinations showed maturing polyclonal plasma cells positive for CD138 and both light chains (Figures 2(e), 2(f) (*κ*), and 2(g) (*λ*)). Negative staining for SV40 and IgG4 in these infiltrates excluded BK virus-related nephropathy and IgG4-related kidney disease, respectively (Figures 2(b) (SV40) and 2(d) (IgG4)). Moreover, negative staining for EBV-encoded small RNA by in situ hybridization excluded posttransplant lymphoproliferative disorder (PTLD) (Figure 2(c)). Thus, we diagnosed the patient with PCAR (Banff 2013 classification: i2, t2, g0, ptc1, v0, ci0, ct0, cg0, cv0, ah1, and aah0). To prevent recurrence of CMV infection, we administered 500 mg mPSL for three days without antithymocyte globulin. The patient's S-Cr level was 1.54 mg/dL and did not increase. Three months later, we performed a second allograft biopsy to evaluate the therapy for rejection. Pathological analysis revealed not only the presence of focal aggressive interstitial mononuclear cell infiltration (Figures 3(b) and 3(c)) but also de novo mild transplant glomerulitis (Figure 3(a)) and focal and moderate peritubular capillaritis (Figure 3(d)), which are characteristics of ABMR. However, neither flow cytometry crossmatch nor donor-specific antibodies (DSAs) for HLA class I or II, including HLA-A, B, C, DR, DQ, and DP, were found in serum samples, and the peritubular capillaries did not show C4d staining by immunohistochemistry. Thus, we diagnosed residual PCAR without ABMR (Banff 2013 classification: i1, t1, g1, ptc2, v0, ci2, ct2, cg0, cv0, ah1, and aah0) and performed additional mPSL pulse therapy alone, using the same regimen as before. The patient's S-Cr level was 1.71 mg/dL and did not increase.

**Figure 4:**
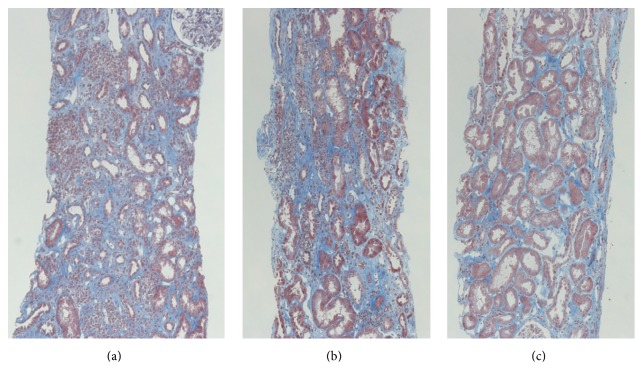
Time-dependent histopathological changes in tubulointerstitial lesions at 3 (a), 6 (b), and 12 months (c) after transplantation ((a–c) Masson's Trichrome, ×100). Light microscopic findings revealed marked regression of plasma cell infiltration at 12 months, compared with at 3 or 6 months after transplantation.

A third allograft biopsy, performed 6 months after the second mPSL series, demonstrated complete disappearance of the PCAR pathological findings (Banff 2013 classification: i1, t0, g0, ptc0, v0, ci2, ct2, cg0, cv0, ah0, and aah0). Figures 4(a)–4(c) demonstrate the gradual improvement of interstitial inflammation of PCAR ((a) first protocol biopsy, (b) second biopsy, and (c) third biopsy). The patient's S-Cr level slightly increased from 1.5 mg/dL to 1.7–2.1 mg/dL during PCAR period, but it was kept at 1.7–2.1 mg/dL after resolution of rejection.

## 3. Discussion

Here we report an unusual case of PCAR diagnosed at an early phase after kidney transplantation which responded well to steroid therapy alone. This case should help address two issues: the risk factors for PCAR development and the clinical and pathological conditions that lead to better graft outcomes for PCAR.

This case developed PCAR after administration of antiviral medications for a refractory CMV infection, including foscarnet, and reduction of immunosuppressive agents. This might suggest a significant influence of immunosuppressive drugs on the development of PCAR. Previous studies have suggested reduced immunosuppressive drug doses, noncompliance with immunosuppressive drug regimens, and female sex as possible risk factors for PCAR [2, 3, 5]. In addition, Lerut et al. reported that noncompliant recipients tend to have greater plasma cell densities among inflammatory cells after allograft rejection compared to compliant recipients, although this difference was not significant (10.25 ± 8.19% versus 8.52 ± 4.23%, *p* = not significant) [12]. Moreover, a recent report showed that a quarter of PCAR patients have a history of discontinued calcineurin inhibitor use after transplantation, associated with economic problems in Pakistan [13]. Therefore, we should monitor the development of PCAR, especially in cases with an unavoidable dosage reduction of immunosuppressive agents.

PCAR commonly develops during a relatively late phase after transplantation, compared with other types of rejection. According to Desvaux et al., PCAR is diagnosed at a median of 187 days after transplantation, with all cases arising during the first year after transplantation [1]. By contrast, we were able to incidentally diagnose the present case with subclinical PCAR during an earlier phase (three months after transplantation), suggesting that an earlier diagnosis might lead to favorable allograft outcomes in PCAR. No reports have identified an association between the time of diagnosis and allograft outcome in PCAR, but several reports have provided suggestive evidence. Meehan et al. reported kidney allograft outcomes for 19 recipients with plasmacytic infiltrates. Excluding three cases diagnosed with PTLD, 62.5% of recipients (10/16) lost their kidney allografts primarily because of acute and chronic rejection. Recipients with graft survival tended to be diagnosed earlier than did those with graft loss (average diagnosis time: 20.3 versus 40.9 months) [4]. Furthermore, among PCAR case reports, recipients with less than moderate interstitial inflammation (Banff classification: i1-2) tended to be diagnosed with PCAR earlier than were those with severe inflammation (Banff classification: i3) (average time 13.5 versus 24.7 months, *p* = 0.21 by Mann–Whitney test; Table 1) [5–11]. While these results suggest the importance of early PCAR diagnosis for prompt therapy and better allograft outcomes, whether an association exists between the timing of PCAR diagnosis and graft survival is still unknown. Further clinical studies are needed to clarify this.

Previous reports have shown PCAR to be refractory to steroid therapy [1–4]. Of the 14 [1] and 2 cases [2] evaluated by previous studies, all episodes of PCAR were steroid resistant. In our case, multiple courses of pulse steroid therapy were needed to resolve PCAR, reflecting this property. Notably, PCAR in our case responded very well to pulse steroid therapy alone. Allograft biopsies did not show findings of ABMR with DSAs, which might influence the effectiveness of steroid therapy alone for PCAR. Among rejection recipients without PCAR, TCMR is often sensitive to steroid therapy [14]. By contrast, ABMR is often resistant to steroid therapy alone, and multiple antirejection therapies have been recommended for ABMR, including IVIG, rituximab, and plasma exchange [15]. Furthermore, a previous report demonstrated steroid sensitivity in a PCAR case negative for C4d immunostaining in peritubular capillaries and without DSAs, as in our case [7]. Moreover, Abbas et al. showed a strong effect of DSAs in PCAR leading to allograft loss (*p* = 0.001 by log-rank test) [13]. These results illustrate better graft outcomes after PCAR without ABMR and suggest that the presence of multiple pathophysiological conditions, including that of ABMR, underlies the discrepancies in the effects of antirejection therapy. Therefore, in considering therapy for PCAR, we should be sure to identify these conditions and, especially, to distinguish between ABMR and non-ABMR.

Although PCAR without ABMR has no consensus therapy today, induction therapy for PCAR without ABMR commonly consists of not only steroid but also multiple immunosuppressant including deoxyspergualin (DSG) and Muromonab-CD3 (OKT3) [5, 6]. In this case, only two times of steroid pulse therapies induced not only the disappearance of PCAR activity without notable allograft dysfunction successfully but also the development of chronic pathological changes with moderate interstitial fibrosis/tubular atrophy (IF/TA). More aggressive antirejection therapy might prevent the progression of IF/TA, which is important cause of allograft rejection, and might improve allograft survival in this case [16]. However, we are strongly concerned about the fact that high degree of immunosuppression could develop severe and refractory infections, deteriorating allograft and patient survival. Therefore, we have carefully chosen the type and dose of immunosuppression therapy for rejection not to develop or recur infections same as this case.

The importance of the histopathological severity of allograft rejection outcomes in PCAR also remains unclear. In the present case, the pathological development of PCAR remained at a moderate level of focally aggressive tubulointerstitial rejection, without vascular rejection (Banff classification: i2, t2, and v0). This relatively low severity of PCAR may have led to the better graft outcome observed in our case. Gärtner et al. suggested that vascular rejection may be involved in poor graft outcomes in PCAR recipients [17]. Moreover, previous case reports of PCAR showed a tendency toward better allograft outcomes in recipients with mild or moderate interstitial infiltration (Banff classification: i1 and i2), compared with those with severe infiltration (i3) (Table 1) [5–11]. In contrast, a previous report of TCMR without PCAR showed that vascular rejection (Banff classification: ARII and ARIII) led to poorer allograft outcomes in recipients, similar to that seen with PCAR, but the severity of interstitial inflammation was not associated with graft survival [18]. These results suggested that plasma cell infiltration in interstitial lesions has a stronger impact on graft survival than does interstitial infiltration by other cell types. However, whether such infiltration of plasma cells itself reflects graft survival remains unclear, and further clinical studies are needed to address the association between the pathological severity of PCAR and graft survival.

In conclusion, we report a case of PCAR without accompanying ABMR which responded well to pulse steroid therapy. Many PCAR cases are resistant to a variety of therapies, but some may have a better prognosis. Frequent allograft biopsies should be used to detect and treat PCAR early. The severity of plasma cell infiltration may be associated with graft outcome. Further studies should be performed to elucidate the pathogenesis and prognosis of PCAR.

## Figures and Tables

**Table 1:** Previous case reports of plasma cell rich acute rejection.

Report	Age/sex	Cadaver/living	Diffuse/focal	Diagnosis after RTx (m)	TG/VR/DSA	ABMR	Medication	Outcome
Chikamoto et al. [5]	16/F	Living	Diffuse	20	−/+/−	−	mPSL, OKT3, DSG	Graft loss
Suzuki et al. [6]	59/M	Cadaver	Unknown	36	−/−/ND	−	mPSL, DSG	Graft loss
Shimizu et al. [7]	45/M	Living	Focal	18	−/−/ND	−	mPSL	Stable
Yoshikawa et al. [8]	37/M	Living	Diffuse	36	−/−/+ (DQ7)	−	mPSL, PEX, IVIG, Rit, DSG	Worsened
Furuya et al. [9]	33/M	Living	Focal	12	−/−/+ (DQ4, 6)	+	mPSL, PEX, IVIG, Rit	Stable
Hasegawa et al. [10]	30/M	Living	Diffuse	18	+/−/+ (DR52)	+	mPSL, IVIG, Rit, OKT3, DSG	Graft loss
Katsuma et al. [11]	56/M	Cadaver	Focal	21	+/−/+ (DR53)	+	mPSL, PEX, IVIG, Rit	Stable
Our case	47/M	Living	Focal	3	−/−/−	−	mPSL	improved

Diffuse infiltration of PCAR was defined as score i3 in Banff classification or described as “Diffuse.” By contrast, less than i2 was described as “Focal.” ND: no data; VR: vascular rejection; RTx: renal transplantation; TG: transplant glomerulitis; DSA: donor-specific antibody; ABMR: antibody-mediated rejection; mPSL: methylprednisolone; PEX: plasma exchange; IVIG: intravenous immunoglobulin; Rit: Rituximab; OKT3: Muromonab-CD3; DSG: deoxyspergualin.
